# The association between body mass index and risk of preoperative oxygenation impairment in patients with the acute aortic syndrome

**DOI:** 10.3389/fendo.2022.1018369

**Published:** 2022-11-17

**Authors:** Chiyuan Zhang, Ruizheng Shi, Guogang Zhang, Hui Bai, Yanfeng Zhang, Lei Zhang, Xuliang Chen, Zuli Fu, Guoqiang Lin, Qian Xu

**Affiliations:** ^1^ Department of Cardiovascular Medicine, the Third Xiangya Hospital, Central South University, Changsha, Hunan, China; ^2^ Department of Cardiovascular Medicine, Xiangya Hospital, Central South University, Changsha, Hunan, China; ^3^ Department of Cardiovascular Surgery, Xiangya Hospital, Central South University, Changsha, Hunan, China

**Keywords:** body mass index, overweight, obesity, acute aortic syndrome, preoperative oxygenation impairment

## Abstract

**Objective:**

The study aimed to determine the relationship between body mass index (BMI) and the risk of acute aortic syndrome (AAS) with preoperative oxygenation impairment.

**Methods:**

A meta-analysis of published observational studies involving BMI and AAS with preoperative oxygenation impairment was conducted. A total of 230 patients with AAS were enrolled for retrospective analysis. All patients were divided into 2 groups (Non-oxygenation impairment group and Oxygenation impairment group). Logistic regression analysis was performed to assess the relation between BMI and the risk of preoperative oxygenation impairment after the onset of AAS. Dose-response relationship curve and subgroup analysis were conducted to test the reliability of BMI as an independent factor of it.

**Results:**

For the meta-analysis, the quantitative synthesis indicated that excessive BMI increased the risk of preoperative oxygenation impairment (OR: 1.30, 95% CI: 1.05-1.60, *P*_heterogeneity_ = 0.001). For the retrospective analysis, a significant association was observed after adjusting for a series of variables. BMI was significantly related to preoperative oxygenation impairment after the onset of AAS (OR: 1.34, 95% CI: 1.15-1.56, *p <*0.001), and compared with normal weight group (18.5 kg/m^2^ ≤ BMI < 23.0 kg/m^2^), the individuals with excessive BMI were at higher risk of preoperative oxygenation impairment for the obese group (BMI ≥ 25 kg/m^2^) (OR: 17.32, 95% CI: 4.03-74.48, *p <*0.001). A J-shape curve in dose-response relationship analysis further confirmed their positive correlation. Subgroup analysis showed that diastolic blood pressure (DBP) ≥ 90mmHg carried an excess risk of preoperative oxygenation impairment in obese patients.

**Conclusion:**

Excessive BMI was an independent risk factor for AAS with preoperative oxygenation impairment.

## Introduction

Acute aortic syndrome (AAS) is a serious cardiovascular disease characterized by urgent onset, rapid progression, and high mortality, and often requires strict management including emergency operation (1). Studies have shown that approximately 50% of AAS patients are complicated with preoperative oxygenation impairment (2), which not only prolongs mechanical ventilation time and hospitalization, but also increases the risk of death and leads to a poorer clinical prognosis (3). Therefore, assessment of the risk factors for clinical outcomes is critical for risk stratification and management of these patients.

Preoperative oxygenation impairment is closely related to ventilation-to-perfusion mismatch and intrapulmonary shunting caused by some pulmonary pathologic changes such as alveolar epithelial and microvascular endothelial damage and immune cells recruitment to the lungs (4), but the definite pathogenesis of AAS with preoperative oxygenation impairment has not been well illustrated. It is currently considered that inflammation is involved in its occurrence and development (5, 6). The damaged aorta releases a large number of cytokines into circulation through intimal rupture, and local vascular inflammation can further develop into excessive systemic inflammation through circulation, which results in multiple organ dysfunction (6). Since the pulmonary capillary bed is an important reservoir of inflammatory cells, the lungs are often the main site of tissue damage by AAS, leading to a hypoxic state in AAS patients (6).

Overweight and obesity are increasingly becoming a medical and socio-economic problem in both developed and developing countries, and body mass index (BMI) calculated from height and weight was used as the measure of excess weight in a wide range of studies (7). In recent years, studies have revealed that excessive BMI is associated with preoperative oxygenation impairment in patients with acute aortic dissection (AD) or intramural hematoma (IMH) (2, 3), and obese AAS patients have higher levels of inflammation and oxidative stress than those with normal weight, suggesting its potential value for risk stratification in AAS patients (8). However, other studies found there is no correlation between excess weight and increased risk of preoperative oxygenation impairment such as hypoxemia in patients with AD (9, 10). Thus, the relationship between BMI and AAS with preoperative oxygenation impairment was poorly defined. In order to clarify inconsistent findings, we conducted a meta-analysis. Moreover, we performed a retrospective study to confirm these findings. We sought to evaluate the influence of excessive BMI on preoperative oxygenation impairment among AAS patients, and provide new clinical evidence for risk stratification of such patients.

## Methods and material

### Study selection and search strategy

The Literatures search followed the Preferred Reporting Items for Systematic Reviews and Meta-Analyses (PRISMA) guidelines (11) and the protocol was registered at the international prospective register of systematic reviews (PROSPERO) with the registration number CRD42022300844. Studies published up to 1 January 2022 were searched for in PubMed, Embase and Web of Science databases and the keywords were: acute aortic syndrome (including AD, IMH, penetrating ulcer) AND oxygenation impairment (including oxygen deficiency, acute respiratory distress syndrome, acute lung injury, hypoxemia) AND preoperative AND body mass index (including obesity, overweight).

All titles and abstracts of studies were screened to select potentially eligible studies, and full texts of those eligible studies were independently reviewed by two investigators (Chiyuan Zhang and Ruizheng Shi). Studies were included if they satisfied the following criteria: (1) studies confirmed to be observational studies; (2) BMI or excess weight (such as obesity) were used as the exposure factors; (3) studies involved the occurrence of AAS with preoperative oxygenation impairment; (4) studies described the value of outcome events with odds ratio (OR) and 95% confidence interval (CI). The related references of the articles that met the requirements above were also included, and duplicated publications were excluded. Our outcome was limited to AAS with preoperative oxygenation impairment by any of these definitions. Disagreements were discussed and solved through consensus.

### Data extraction and quality assessment

We extracted the following information from each included study: first author’s surname, publication year, country, ethnicity, study size, sex, number of cases, diagnostic criteria of preoperative oxygenation impairment, BMI, BMI categories, OR value with the corresponding 95% CIs and adjustment factors in the multivariable analysis. These data were independently extracted based on selection criteria. The Newcastle-Ottawa Scale (NOS) score was used to evaluate the quality of those observational studies (including case-control and cohort studies) involved (12), and studies with a score of 6 or greater were assigned as high-quality studies.

### Clinical study population

We retrospectively enrolled AAS patients admitted to the Department of Cardiovascular Surgery at Xiangya Hospital from December 2018 to December 2020. The diagnosis of AAS was made by contrast-enhanced computed tomography (CT) of the aorta. Patients with the following conditions were excluded: those aged < 18 years or > 80 years, those who had a clear etiology such as iatrogenic aortic disease, secondary to cardiac surgery or a history of chronic AD or IMH, those with chronic diseases in lung, liver, kidney or malignant tumor, those with cardiac arrest, cardiac tamponade, heart failure, hypotension or shock on admission. The study was approved by the ethics committee of Xiangya Hospital (approval number 202101003, Date: January 15, 2021), and written informed consent was waived given the retrospective nature of the study.

### Collection and definition of clinical variables

We collected patients’ data including age, gender, BMI, current smoking, vital signs (systolic blood pressure, diastolic blood pressure, heart rate, etc.), comorbidities (hypertension, diabetes mellitus, fatty liver, etc.), laboratory tests (white blood cell count, hemoglobin, platelet, creatinine, etc.), echocardiographic and CT scan findings on admission. Preoperative oxygenation impairment was defined as an arterial oxygen tension (P_aO2_)/inspiratory oxygen fraction (F_iO2_) ratio ≤ 200 on admission (3). All patients were divided into 2 groups based on the preoperative oxygenation impairment. Besides, in the stratified analysis, due to the differences in body size between Asians and Europeans, patients were further subdivided as: normal weight (18.5 kg/m^2^ ≤ BMI < 23.0 kg/m^2^), overweight (23.0 kg/m^2^ ≤ BMI < 25.0 kg/m^2^) and obesity (BMI ≥ 25 kg/m^2^) following the Asian criteria (13, 14). AAS is defined as classic acute AD with a patent false lumen and IMH.

### Statistical analysis

For the meta-analysis, we used combined OR and 95% CI to determine the association between BMI and the risk of preoperative oxygenation impairment after the onset of AAS. The *Q* statistic and *I^2^* statistics were utilized to assess the heterogeneity among the included studies, and data were analyzed with a random effect model. To investigate the effect of potential confounders, subgroup analysis was performed based on the available characteristics of these studies and sensitivity analysis by omitting one study at each time. Publication bias was assessed with Begg’s funnel plot test.

For the retrospective analysis, the continuous variables with normally distributed distributions are presented as mean ± SD, and the non-normally distributed continuous variables as median and interquartile range (IQR). The categorical variables were presented by number and percentage. We compared 2 variables using the Student *t* test or Mann-Whitney U test and 3 variables using the One-way ANOVA test or Mann-Whitney U test for continuous data and the chi-squared test for categorical data as appropriate. We used both univariate and multivariate logistic regression models to evaluate the relationship between BMI and AAS with preoperative oxygenation impairment. Considering the possibility of impact of other known confounding factors (age, gender, Stanford classification, current smoking, etc.), we also conducted a subgroup analysis according to these factors and a dose-response relationship analysis to test the reliability of this association.

A two-sided *p* value < 0.05 was considered statistically significant and all statistical analyses were performed by STATA 12.0 and R 4.0.3.

## Results

### Literature search and characteristics of the included studies

A flowchart of the study selection process was shown in **Supplementary Figure 1**. A total of 101 articles were found in PubMed (n = 22), Embase (n = 41) and Web of Science (n = 38). Then, a list of 8 article was selected after eliminating duplicate records (n = 26) and ineligible ones (n = 67) according to the title and/or abstracts screen. Ultimately, 6 articles were included in the quantitative analysis by removing 2 articles after full-text review. **Supplementary Table 1** summarized the characteristics of the included studies. In detail, these studies involved 1244 patients with AAS, and the preoperative oxygenation impairment cases ranged from 21 to 235. Apart from one study conducted in Japan, others were performed in China. Diagnostic criteria of preoperative oxygenation impairment in these studies are as follows: 3 studies used P_aO2_/F_iO2_ ratio ≤ 200, 2 studies used P_aO2_/F_iO2_ ratio ≤ 300, 1 study used the concept of acute lung injury without clarifying the value of P_aO2_/F_iO2_. Besides, according to the NOS criteria, the score of these studies was 6 to 8, which was presented in **Supplementary Figure 2**.

### Quantitative synthesis and analysis

**Figure 1:**
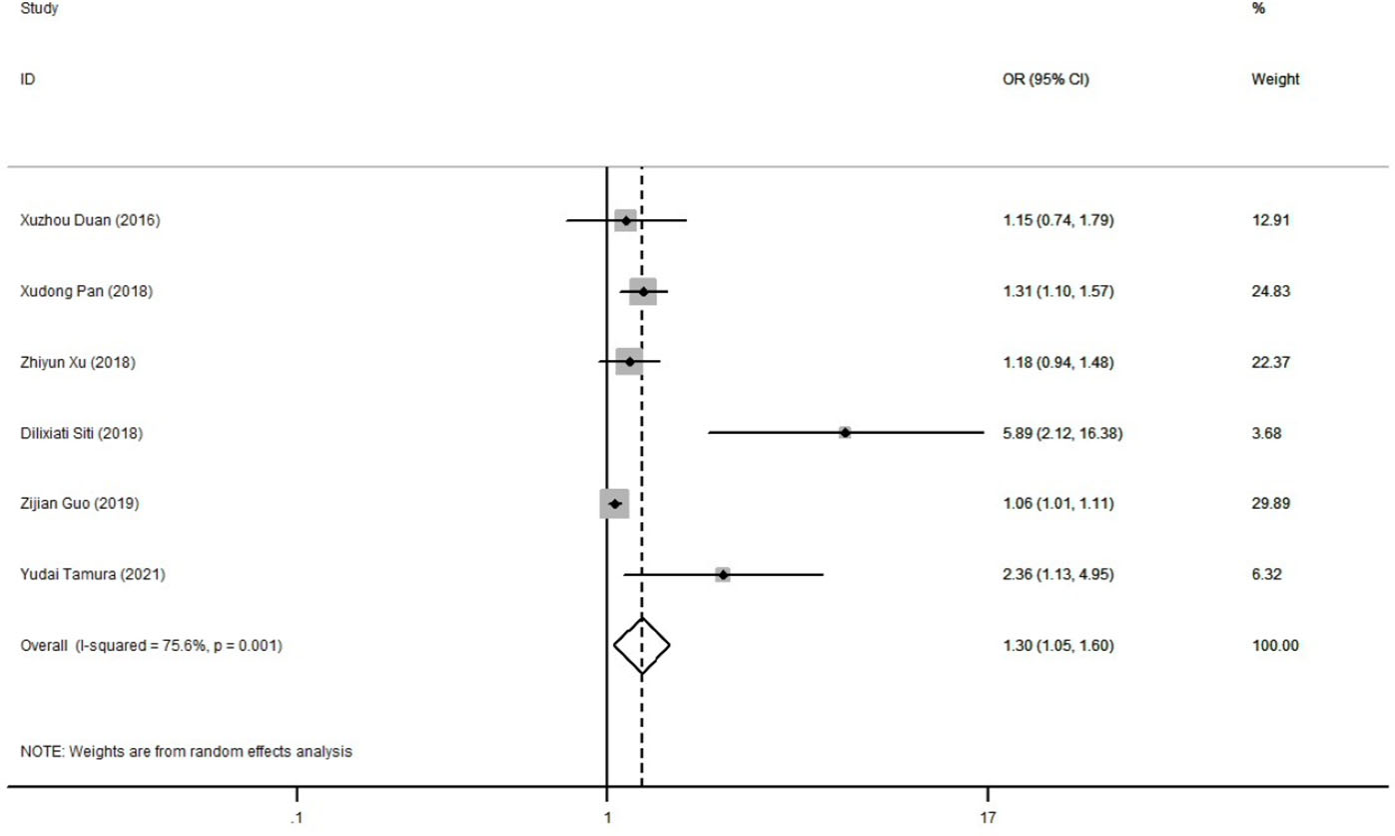
Forest plot of the risk of preoperative oxygenation impairment after the onset of AAS. Gray squares indicate the OR in each study, transparent diamond indicates the combined OR in all studies, and horizontal lines represent the 95% CI. AAS, acute aortic syndrome; OR, odds ratio; CI, confidence interval.

The 6 case-control studies were included in the meta-analysis. A pooled summary showed that excessive BMI had a higher the risk of preoperative oxygenation impairment after the onset of AAS (**Figure 1**). The combined ORs (95% CI) were 1.30 (1.05-1.60), and the heterogeneity in these studies was relatively high (*I^2^* = 75.6%, *P* = 0.001).

To explore the source of heterogeneity of the result above, subgroup analysis and sensitivity analysis were conducted subsequently. **Supplementary Table 2** summarized the results of the subgroup analysis. When stratified by Stanford classification, there was a higher risk of preoperative oxygenation impairment in Stanford type B AAS patients with excess weight. In the stratified analysis by different data forms for BMI, studies choosing BMI as continuous data had a greater risk of preoperative oxygenation impairment than those using BMI as categorical data. Moreover, the sample size was also significantly related to the risk of preoperative oxygenation impairment for AAS patients with excess weight. The sensitivity analysis revealed that none of the individual studies had a large influence on the pooled result (**Supplementary Figure 3**). In addition, Begg’s test indicated no publication bias (*P* = 0.260), but the funnel plot was asymmetric, so its possibility remains to be considered (**Supplementary Figure 4**).

### Baseline characteristics of AAS patients

A total of 285 AAS patients were enrolled from the Xiangya hospital for this retrospective study. Among them, 55 patients were excluded due to age < 18 years or > 80 years (n = 3), a history of chronic AD or IMH (n = 42), chronic lung disease (n = 6), chronic liver disease (n = 10), chronic kidney disease (n = 8), malignant tumor (n = 1), cardiac arrest (n = 1), cardiac tamponade (n = 2), hypotension or shock (n =3) (**Supplementary Figure 5**). Thus, 230 AAS patients were ultimately recruited for further analysis. There were 72 AAS patients with preoperative oxygenation impairment (Oxygenation impairment group) and 158 controls (Non-oxygenation impairment group), and the baseline characteristics of the patients were presented in **Table 1**. In detail, the patients in the oxygenation impairment group were younger and had a higher BMI (all *p* < 0.05). Obesity, fatty liver and hypertension were more common among them (all *p* < 0.05), while coronary artery disease (CAD) was more common in the Non-oxygenation impairment group (*p* < 0.05). In laboratory and imaging examination, patients with preoperative oxygenation impairment had higher levels of diastolic blood pressure (DBP), creatinine (Cr), and P_aO2_/F_iO2_ ratio (all *p* < 0.05), and were less likely to have Stanford type A AAS and aortic regurgitation than non-oxygenation impairment patients (all *p* < 0.05). After hospitalization, the oxygenation impairment group received more vasodilators (*p* < 0.01). Then, stratified analysis was performed based on BMI, which was shown in **Table 2**. The proportion of males and P_aO2_/F_iO2_ ratio was positively related to BMI increase (all *p* < 0.05). In addition, the fatty liver, hypertension, preoperative oxygenation impairment, and a higher level of DBP, neutrophile (NE), and C reactive protein (CRP) were more common in excessive BMI groups (all *p* < 0.05).

**Table 1:** Baseline characteristics of study participants.

	Non-oxygenation impairment (n=158)	Oxygenation impairment (n=72)	*p* values
Age (years)	54.46 ± 13.21	50.01 ± 10.75	0.013
Sex, male (%)	117 (74.1)	60 (83.3)	0.121
BMI (kg/m^2^)	23.65 (22.04-26.15)	26.63 (25.49-29.39)	<0.001
BMI, n (%)			<0.001
18.5 ≤ BMI < 23	65 (41.1)	4 (5.6)	
23 ≤ BMI < 25	37 (23.4)	8 (11.1)	
BMI ≥ 25	56 (35.4)	60 (83.3)	
Current smoking, n (%)	77 (48.7)	43 (59.7)	0.122
Medical history			
Fatty liver, n (%)	39 (34.8)	34 (57.6)	0.004
Hypertension, n (%)	107 (67.7)	60 (83.3)	0.014
CAD, n (%)	17 (10.8)	2 (2.8)	0.041
Diabetes, n (%)	6 (3.8)	1 (1.4)	0.324
Vital signs on admission			
SBP (mmHg)	142.00 (123.00-160.00)	147.50 (129.50-171.50)	0.078
DBP (mmHg)	70.00 (62.00-80.00)	76.50 (65.00-89.25)	0.018
Heart rate (/min)	75.50 (65.75-92.25)	82.5 (71.00-92.00)	0.149
Laboratory data on admission			
WBC (×10^9^/L)	11.20 (8.60-13.75)	11.95 (9.53-15.00)	0.076
NE (×10^9^/L)	8.80 (6.65-11.50)	10.00 (7.03-12.93)	0.144
Hb (g/L)	130.00 (120.00-139.00)	134.50 (123.00-145.00)	0.084
PLT (×10^9^/L)	168.00 (130.50-213.50)	192 (137.00-226.00)	0.151
D-dimer (μg/mL)	1.55 (0.76-2.78)	1.36 (0.50-2.84)	0.465
Cr (μmol/l)	91.60 (73.95-106.85)	99.65 (84.70-127.65)	0.004
CRP (mg/l)	42.25 (8.86-92.53)	47.50 (15.30-78.90)	0.866
P_aO2_/F_iO2_ ratio	341.00 (269.25-483.50)	171.00 (143.75-185.00)	<0.001
Ultrasound and CT findings on admission			
Stanford classification			0.036
Type A, n (%)	118 (74.7)	44 (61.1)	
Type B, n (%)	40 (25.3)	28 (38.9)	
AD, n (%)	136 (88.3)	66 (91.7)	0.446
IMH, n (%)	33 (21.4)	12 (16.7)	0.404
LVEF (%)	60.00 (56.00-67.00)	60.00 (56.00-66.00)	0.532
LVEDD (mm)	50.00 (45.00-53.50)	50.00 (47.00-54.00)	0.547
Aortic regurgitation, n (%)	127 (89.4)	48 (73.8)	0.004
Pleural effusion, n (%)	32 (22.7)	19 (28.8)	0.344
Medications on admission			
Vasodilators, n (%)	111 (70.3)	63 (87.5)	0.005

Data are presented as mean ± SD, n (%), or medians (interquartile ranges). BMI, body mass index; CAD, coronary artery disease; SBP, systolic blood pressure; DBP, diastolic blood pressure; WBC, white blood cell; NE, neutrophile; Hb, hemoglobin; PLT, platelet; Cr, creatinine; CRP, C reactive protein; P_aO2_/F_iO2_, arterial oxygen tension/inspiratory oxygen fraction; AD, aortic dissection; IMH, intramural hematoma; LVEF, left ventricular ejection fraction; LVEDD, left ventricular end-diastolic dimension.

**Table 2:** Stratified analysis of baseline characteristics according to BMI.

	18.5 ≤ BMI < 23 (n=69)	23 ≤ BMI < 25 (n=45)	BMI ≥ 25 (n=116)	*p* values
Age (years)	55.26 ± 14.63	53.62 ± 10.34	51.54 ± 12.06	0.181
Sex, male (%)	45 (65.2)	35 (77.8)^a^	97 (83.6)^bc^	0.016
Current smoking, n (%)	30 (43.5)	23 (51.1)	67 (57.8)	0.169
Medical history				
Fatty liver, n (%)	6 (13.0)	10 (27.8)	57 (64.0)^bc^	<0.001
Hypertension, n (%)	38 (55.1)	36 (80.0)^a^	93 (80.2)^c^	<0.001
CAD, n (%)	7 (10.1)	3 (6.7)	9 (7.8)	0.774
Diabetes, n (%)	3 (4.3)	2 (4.4)	2 (1.7)	0.501
Vital signs on admission				
SBP (mmHg)	142.00 (120.00-162.00)	142.00 (118.00-157.50)	145.50 (130.00-168.75)	0.136
DBP (mmHg)	65.00 (62.00-78.00)	69.00 (62.00-80.00)	76.00 (65.00-84.75)^c^	0.010
Heart rate (/min)	75.00 (65.00-91.00)	82.00 (66.50-92.00)	82.00 (68.00-94.75)	0.331
Laboratory data on admission				
WBC (×10^9^/L)	11.3 (8.45-13.78)	10.6 (8.30-12.15)	11.8 (9.60-14.80)	0.090
NE (×10^9^/L)	8.80 (6.53-11.70)	8.40 (5.90-10.35)	9.85 (7.13-12.60)^b^	0.039
Hb (g/L)	124.00 (112.25-138.00)	129.00 (123.00-135.50)	136.00 (123.00-145.00)^c^	0.002
PLT (×10^9^/L)	162.00 (141.25-202.75)	182.00 (130.50-231.00)	174.50 (131.50-214.75)	0.613
D-dimer (μg/mL)	1.66 (0.83-3.06)	1.14 (0.47-2.67)	1.52 (0.78-2.81)	0.317
Cr (μmol/l)	85.30 (70.70-102.08)	91.60 (77.15-123.70)	98.35 (80.93-113.68)^c^	0.024
CRP (mg/l)	19.45 (3.96-76.80)	42.80 (9.96-117.00)	66.65 (15.38-91.93)^c^	0.022
P_aO2_/F_iO2_ ratio	424.00 (287.50-572.50)	294.00 (209.00-440.50)^a^	200.00 (161.00-293.75)^bc^	<0.001
Ultrasound and CT findings on admission				
Stanford classification				0.901
Type A, n (%)	50 (72.5)	31 (68.9)	81 (69.8)	
Type B, n (%)	19 (27.5)	14 (31.1)	35 (30.2)	
AD, n (%)	56 (82.4)	41 (93.2)	105 (92.1)	0.078
IMH, n (%)	17 (25.0)	7 (15.9)	21 (18.4)	0.426
LVEF (%)	60.00 (56.00-66.25)	63.00 (57.00-69.00)	60.00 (56.00-65.00)	0.418
LVEDD (mm)	48.00 (44.00-52.00)	50.00 (45.00-57.00)	51.50 (47.00-54.00)^c^	0.021
Aortic regurgitation, n (%)	58 (92.1)	31 (79.5)	86 (81.9)	0.132
Pleural effusion, n (%)	18 (28.6)	8 (20.5)	25 (23.8)	0.631
Medications on admission				
Vasodilators, n (%)	50 (52.2)	29 (64.4)	95 (81.9)	0.052
Oxygenation impairment, n (%)	4 (5.8)	8 (17.8)	60 (51.7)^bc^	<0.001

Data are presented as mean ± SD, n (%), or medians (interquartile ranges).

a *P* < 0.05 for 18.5 ≤ BMI < 23 group vs 23 ≤ BMI < 25 group;

b *P* < 0.05 for 23 ≤ BMI < 25 group vs BMI ≥ 25 group;

c *P* < 0.05 for 18.5 ≤ BMI < 23 group vs BMI ≥ 25 group;

BMI, body mass index; CAD, coronary artery disease; SBP, systolic blood pressure; DBP, diastolic blood pressure; WBC, white blood cell; NE, neutrophile; Hb, hemoglobin; PLT, platelet; Cr, creatinine; CRP, C reactive protein; P_aO2_/F_iO2_, arterial oxygen tension/inspiratory oxygen fraction; AD, aortic dissection; IMH, intramural hematoma; LVEF, left ventricular ejection fraction; LVEDD, left ventricular end-diastolic dimension.

### The association between BMI and AAS with oxygenation impairment

**Figure 2:**
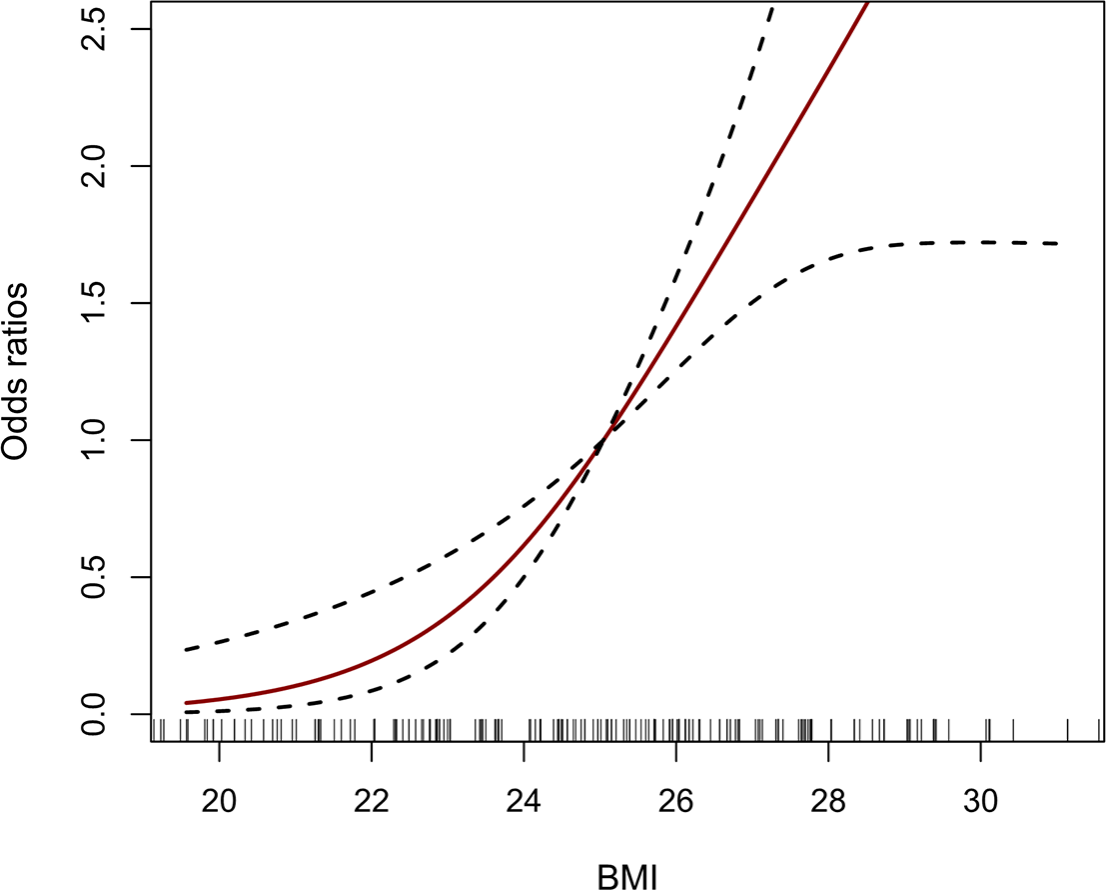
The dose-response relationship between BMI and AAS with preoperative oxygenation impairment. The vertical black bars represent individual BMI values. The solid red line and the dash black line represent the estimated OR and its 95% CI. BMI, body mass index; AAS, acute aortic syndrome; OR, odds ratio; CI, confidence interval.

To explore the relationship between BMI and the risk of AAS with preoperative oxygenation impairment, a logistic regression analysis was conducted. A significant association was observed in the study after adjusting for age, gender, fatty liver, hypertension, CAD, DBP, Cr, Stanford classification, aortic regurgitation and vasodilators. As illustrated in **Table 3**, by multiple logistic regression analysis, BMI was independently related to preoperative oxygenation impairment after the onset of AAS (OR: 1.34, 95% CI: 1.15-1.56, *p <*0.001), and compared with the normal weight group (18.5 kg/m^2^ ≤ BMI < 23.0 kg/m^2^), the individuals with excessive BMI were at higher risk of preoperative oxygenation impairment for the obese group (BMI ≥ 25 kg/m^2^) (OR: 17.32, 95% CI: 4.03-74.45, *p <*0.001). Besides, the dose-response relationship presented a J-shaped curve, that was, the risk of AAS with preoperative oxygenation impairment increased with the increased BMI (**Figure 2**).

**Table 3:** The association between BMI and AAS with preoperative oxygenation impairment in univariate and multivariate logistic regression analysis.

	Univariate		Multivariate	
	OR (95% CI)	*p* values	OR (95% CI)	*p* values
BMI	1.40 (1.25-1.56)	<0.001	1.34 (1.15-1.56)	<0.001
18.5 ≤ BMI < 23	Reference	–	Reference	–
23 ≤ BMI < 25	3.51 (0.99-12.47)	0.052	4.10 (0.82-20.48)	0.085
BMI ≥ 25	17.41 (5.95-50.93)	<0.001	17.32 (4.03-74.48)	<0.001

BMI, body mass index; AAS, acute aortic syndrome; OR, odds ratio.

For interaction analysis, the study participants were divided into different subgroups according to gender, age, Stanford classification, current smoking, fatty liver, hypertension, CAD, diabetes, vasodilators, DBP, D-dimer, fibrinogen, etc. The results showed no interaction in most strata (*p* for interaction = 0.058-0.921). Only obese AAS patients with a DBP ≥ 90mmHg had an excess risk of preoperative oxygenation impairment (OR: 37.40, 95% CI: 3.84-364.57, *p <*0.05) (**Table 4**).

**Table 4:** Interaction analysis of the association with BMI and AAS with preoperative oxygenation impairment.

	No. of patients	BMI levels (kg/m^2^)			*p* for interaction
		18.5-23	23-25	≥ 25	
Gender					0.136
Male	177	ref	3.58 (0.65-19.71)	25.90 (5.94-112.98)	
Female	53	ref	4.71 (0.65-34.18)	6.42 (1.15-35.90)	
Age, years					0.004
< 60	163	ref	/	/	
≥ 60	67	ref	0.69 (0.07-7.07)	2.50 (0.66-9.51)	
Stanford classification					<0.001
Type A	162	ref	3.35 (0.89-12.61)	7.91 (2.50-24.08)	
Type B	68	ref	/	/	
AD					0.704
No	24	ref	/	13.75 (1.21-156.65)	
Yes	202	ref	4.28 (1.06-17.30)	19.43 (5.71-66.13)	
IMH					0.525
No	181	ref	4.41 (1.08-17.98)	17.82 (5.18-61.29)	
Yes	45	ref	/	17.6 (1.96-157.94)	
Current smoking					0.324
No	110	ref	5.44 (0.96-30.92)	15.07 (3.26-69.63)	
Yes	120	ref	2.10 (0.32-13.75)	18.35 (4.04-83.36)	
Fatty liver					0.178
No	98	ref	3.46 (0.58-20.42)	27.77 (5.68-135.81)	
Yes	73	ref	3.33 (0.277-40.29)	5.18 (0.57-47.16)	
Hypertension					0.921
No	63	ref	/	/	
Yes	167	ref	2.05 (0.55-7.72)	9.47 (3.11-28.81)	
CAD					0.058
No	211	ref	2.90 (0.79-10.62)	17.82 (6.04-52.61)	
Yes	19	ref	/	/	
Diabetes					0.918
No	223	ref	3.54 (1.00-12.61)	16.63 (5.67-48.76)	
Yes	7	ref	/	/	
Vasodilators					0.546
No	56	ref	/	/	
Yes	174	ref	3.00 (0.77-11.70)	14.51 (4.84-43.55)	
SBP, mmHg					0.552
< 140	93	ref	1.50 (0.19-11.64)	14.79 (3.13-69.90)	
≥ 140	137	ref	6.00 (1.10-32.60)	20.09 (4.50-89.58)	
DBP, mmHg					0.044
< 90	192	ref	4.36 (1.08-17.62)	15.18 (4.43-51.99)	
≥ 90	38	ref	/	37.40 (3.84-364.57)	
D-dimer, μg/mL					0.427
< 0.5	40	ref	0.64 (0.07-5.61)	7.00 (1.10-44.61)	
≥ 0.5	188	ref	6.58 (1.24-34.80)	27.92 (6.45-120.83)	
Fibrinogen, g/L					0.078
< 4	166	ref	10.20 (1.99-52.24)	27.37 (6.26-119.68)	
≥ 4	63	ref	11.16 (2.05-49.93)	6.93 (1.34-35.99)	
ESR, mm/h					0.266
< 20	65	ref	20.00 (1.74-229.49)	22.35 (2.70-184.79)	
≥ 20	73	ref	2.04 (0.30-13.85)	8.97 (1.81-44.47)	
CRP, mg/l					0.502
< 8	18	ref	11.00 (0.35-345.06)	33.00 (1.56-697.96)	
≥ 8	74	ref	1.94 (0.36-10.43)	5.90 (1.48-23.44)	
IL-1β, pg/ml					0.454
< 5.0	43	ref	5.50 (0.61-49.54)	3.93 (0.71-21.75)	
≥ 5.0	52	ref	1.40 (0.08-25.14)	8.75 (0.99-77.19)	
TNF-α, pg/ml					0.904
< 8.1	33	ref	/	/	
≥ 8.1	49	ref	1.87 (0.24-14.65)	3.67 (0.84-16.04)	
IL-10, pg/ml					0.215
< 9.1	70	ref	1.64 (0.28-9.58)	2.75 (0.67-11.32)	
≥ 9.1	24	ref	/	/	
Aortic regurgitation					0.609
No	32	ref	1.33 (0.09-20.11)	11.2 (1.00-125.64)	
Yes	175	ref	3.53 (0.78-15.89)	15.94 (4.63-54.92)	
Pleural effusion					0.846
No	110	ref	1.83 (0.24-14.13)	14.79 (3.23-67.79)	
Yes	78	ref	2.86 (0.46-17.81)	13.75 (2.81-67.41)	

BMI, body mass index; AAS, acute aortic syndrome; AD, aortic dissection; IMH, intramural hematoma; CAD, coronary artery disease; SBP, systolic blood pressure; DBP, diastolic blood pressure; ESR, erythrocyte sedimentation rate; CRP, C reactive protein; IL-1β, Interleukin-1β; TNF-α, Tumor necrosis factor-α; IL-10, Interleukin-10.

## Discussion

In our study, we explored the relationship between BMI and preoperative oxygenation impairment after the onset of AAS. Our meta-analysis demonstrated that excessive BMI increased the risk of AAS with preoperative oxygenation impairment. Similarly, our retrospective study further confirmed this correlation above. Multivariate logistic analysis suggested that both excessive BMI could be independent risk factors for preoperative oxygenation impairment in AAS patients. A dose-response relationship curve showed that BMI was positively correlated with the incidence of AAS with preoperative oxygenation impairment. Subgroup analysis indicated that DBP ≥ 90mmHg carried an excess risk of preoperative oxygenation impairment in obese patients with AAS. Our study provided new clinical evidence for risk stratification of AAS patients with preoperative oxygenation impairment.

Preoperative oxygenation impairment is a serious complication that occurs in patients with AAS, which is not only life-threatening but also prolongs the length of ventilator support and intensive care unit (ICU) stay (15). In recent years, with the increasing incidence of the complication, studies have focused on its relationship with BMI, but the results are controversial. For instance, Tamura Y and his colleagues found in a retrospective study of 224 Stanford type B AAS patients that obesity (defined as BMI ≥ 25 kg/m^2^) was an independent risk factor of preoperative oxygenation impairment (3). Similarly, Pan X and his colleagues demonstrated that excessive BMI was significantly related to the occurrence of preoperative oxygenation impairment in patients with Stanford type A AD (2). However, two studies from China (9, 16) respectively revealed that there was no significant association between excessive BMI and the increased risk of preoperative oxygenation impairment in acute AD patients. These controversial results may be stemmed from the differences in the diagnostic criteria of oxygenation impairment, the dissimilarities in patients’ inclusion criteria, and the different adjustments for identifying the risk factors of AAS with preoperative oxygenation impairment

In the present study, we reviewed 6 studies for meta-analysis. Based on the NOS scores, the included studies were of high quality (a score of 6-8), suggesting their reliable results. The quantitative synthesis indicated that BMI was independently related to oxygenation impairment after the onset of AAS. Similar results were observed in the subgroup analysis of the Stanford classification and different data forms for BMI. Furthermore, we retrospectively recruited 230 individuals in our center and found that preoperative oxygenation impairment occurred in approximately one-third of patients with AAS. These patients were younger, had higher BMI, DBP and Cr, more fatty liver, hypertension, used vasodilators, and had less Stanford type A AAS, CAD, and aortic regurgitation. When we further stratified all subjects according to the BMI, a significant positive correlation between BMI and P_aO2_/F_iO2_ ratio was identified, and preoperative oxygenation impairment was more common in obese patients with AAS. We further found that overweightness is an independent risk factor of AAS with preoperative oxygenation impairment, which confirms the results of our meta-analysis. Also, the dose-response analysis and subgroup analysis proved its reliability and potential value for prediction. Recent studies have shown that the AAS complicated by oxygenation impairment is closely related to inflammation, and it is believed that the progression of local vascular inflammation caused by intima tear and hematoma formation in AAS plays a vital role, which can develop into systemic inflammatory reaction and result in acute lung injury(17). However, few studies have investigated its pathogenesis in obese patients. Obese patients have abundant adipose tissue, but the vascular system in the tissue is underdeveloped so adipocytes are prone to hypoxia (18). Continuous over-nutrition eventually leads to a state of chronic hypoxia within the adipose tissue in obese patients (18). Also, adipose tissue can release a variety of cytokines including inflammatory factors into circulation (19), and hypoxia has been demonstrated to be one of the most potent stimuli for the release of a series of inflammatory factors such as interleukin-1β (IL-1β), tumor necrosis factor-α (TNF-α) and interleukin-6 (IL-6), and activating pro-inflammatory signaling pathways (20, 21). Thus, obese patients have subclinical chronic inflammation, leading to a predisposition to a severe respiratory inflammatory response and oxygenation impairment (21). It is reported that obese patients with AAS showed an elevated IL-1β, TNF-α, and IL-6 (8), while our study also revealed that these patients had a higher level of NE and CRP (*P* = 0.039, 0.022), suggesting a more severe inflammatory response in such patients. These inflammatory reactants may directly destroy pulmonary vascular endothelial cells through circulation, resulting in pulmonary dysfunction and oxygenation impairment (17), and hypoxia can further stimulate adipocytes to release inflammatory cytokines, creating a vicious cycle. In addition, obesity is a significant factor for hypoxemia and ventilation in the ICU, (22). The high-fat content in the pleura or chest walls of obese patients can limit thoracic breathing and diaphragmatic activity and reduce respiratory resistance and airway resistance (22, 23).

The result of the subgroups analysis showed no interaction in most strata, which proved that obesity was a reliable independent risk factor for AAS with preoperative oxygenation impairment. We also found that obese AAS patients with DBP ≥ 90mmHg had an excess risk of oxygenation impairment. It is well known that one of the main treatments for AAS is the antihypertensive therapy, to decrease the shear stress on the aortic wall and reduce the size of the tear in the false lumen (24). Thus, the higher blood pressure in AAS, the higher the risk of aortic rupture and/or other complications such as visceral and peripheral ischemia, which can promote oxygenation impairment (25). Besides, DBP is an indicator of peripheral vascular resistance. A significantly increased DBP in patients with AAS can lead to organ malperfusion including lung and may contribute to the ventilation-blood flow mismatch and a state of hypoxia. Perhaps, it could explain the reason why DBP ≥ 90mmHg had an excess risk of oxygenation impairment in obese patients with AAS. Furthermore, our result suggested that more severe measures should be taken in this situation.

It is the first time to estimate the association between different BMI groups and AAS with preoperative oxygenation impairment. Together with previous studies, our results supported the potential value of BMI as an indicator for risk stratification and obesity as an independent risk factor of preoperative oxygenation impairment in AAS patients, which provided potent clinical evidence for the prevention and management of such patients. The limitations of this study are listed as follows: firstly, the inclusion of case-control studies and significant heterogeneity in the meta-analysis increased bias in the results; secondly, our study included a retrospective single-center analysis and its sample size was relative small, which might not be universally representative. More large-scale prospective studies need to validate the present results in the future.

In conclusion, our findings are consistent with the general consensus that excessive BMI is an independent risk factor for AAS with preoperative oxygenation impairment. AAS patients who have a BMI of 25 or greater are at increased risk of preoperative oxygenation impairment.

## Data availability statement

The datasets presented in this study can be found in online repositories. The names of the repository/repositories and accession number(s) can be found in the article/**Supplementary Material**.

## Ethics statement

The studies involving human participants were reviewed and approved by the ethics committee of Xiangya Hospital. Written informed consent for participation was not required for this study in accordance with the national legislation and the institutional requirements.

## Author contributions

QX and GL conceived the study. CZ reviewed publications, extracted the data of eligible studies for the meta-analysis, performed the statistical analysis and wrote the first draft of the paper. RS reviewed publications and extracted the data of eligible studies for the meta-analysis. GZ and HB extracted the data of eligible studies for the meta-analysis. YZ and LZ recruited the AAS patients with and without oxygenation impairment and collected their clinical data. XC and ZF performed the statistical analysis. All authors contributed to the article and approved the submitted version.

## Funding

This study was supported by two grants from the Hunan Provincial Natural Science Foundation of China (Hunan, China; Grant No. 2021JJ41041 and No. 2022JJ70158)

## Acknowledgments

We would like to thank Yuwei Wu for statistical assistance.

## Conflict of interest

The authors declare that the research was conducted in the absence of any commercial or financial relationships that could be construed as a potential conflict of interest.

## Publisher’s note

All claims expressed in this article are solely those of the authors and do not necessarily represent those of their affiliated organizations, or those of the publisher, the editors and the reviewers. Any product that may be evaluated in this article, or claim that may be made by its manufacturer, is not guaranteed or endorsed by the publisher.
