# Supplementary material for: The association between body mass index and risk of preoperative oxygenation impairment in patients with the acute aortic syndrome
Source: Front Endocrinol (Lausanne). 2022 Nov 17;13:1018369. doi: 10.3389/fendo.2022.1018369 (PMC9712723; doi:10.3389/fendo.2022.1018369)
Supplement: Supplementary file 1 [file DataSheet_1.docx]

**Figure legends**

**Supplementary Figure 1.**


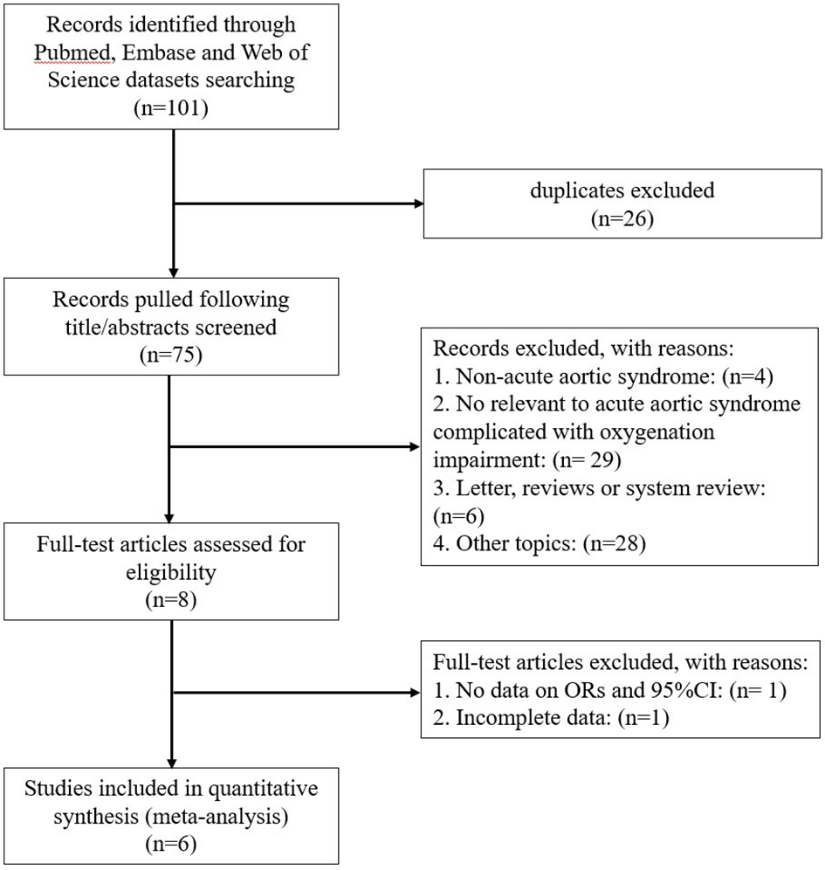


Supplementary Figure 1. Flowchart of study selection.

**Supplementary Figure 2.**


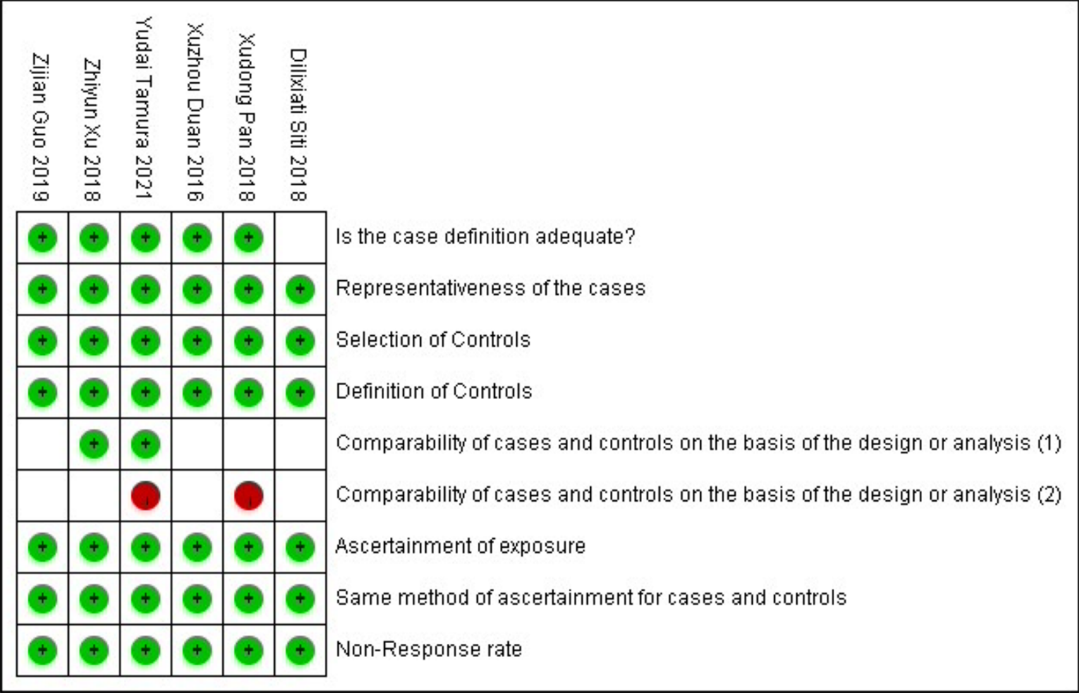


Supplementary Figure 2. Summary of risk of bias in the included studies.

**Supplementary Figure 3.**


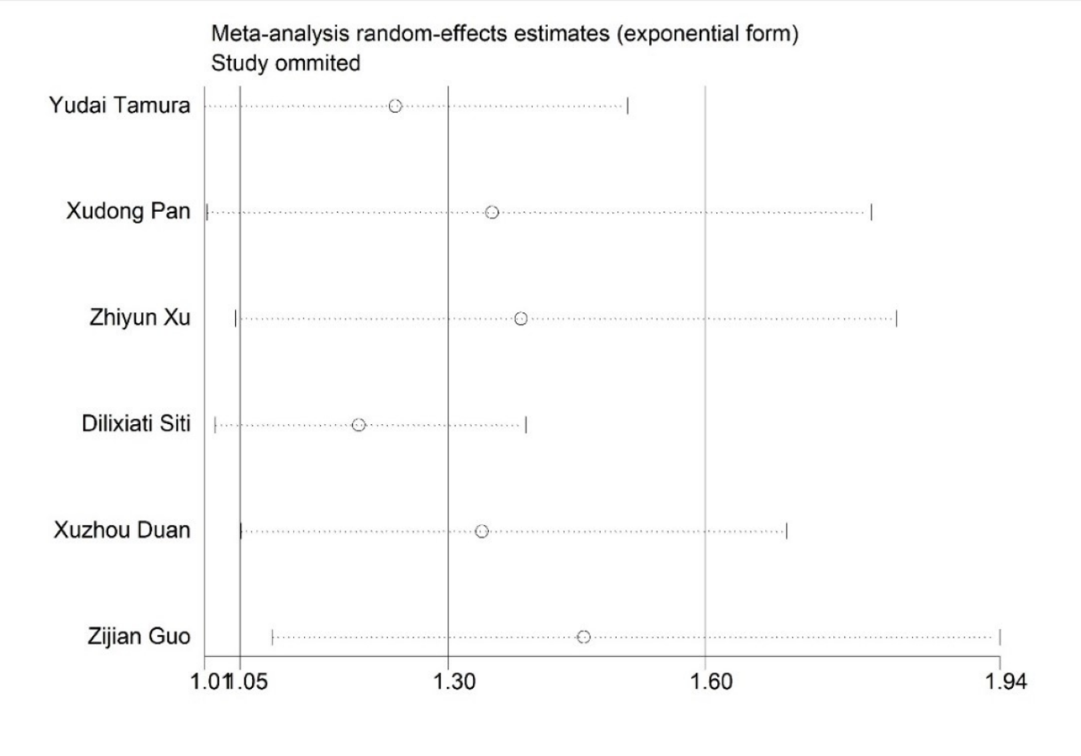


Supplementary Figure 3. The leave-one-out sensitivity analysis from the included studies.

**Supplementary Figure 4.**


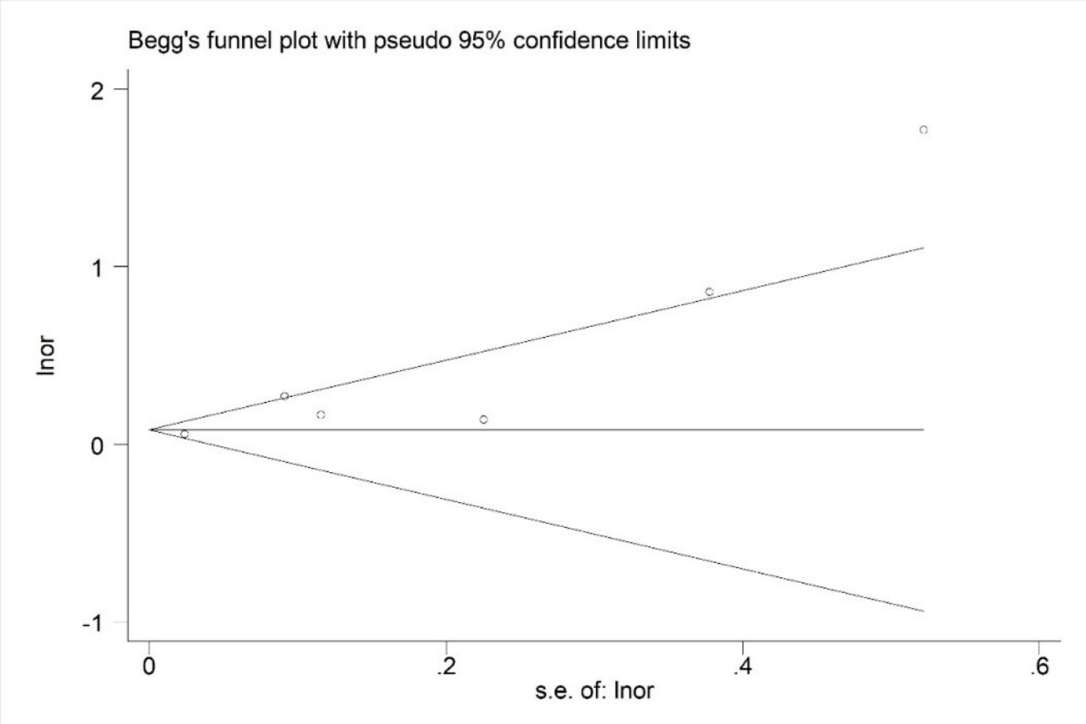


Supplementary Figure 4. Publication bias in funnel plot of BMI and AAS with preoperative oxygenation impairment. BMI: body mass index; AAS: acute aortic syndrome.

**Supplementary Figure 5.**


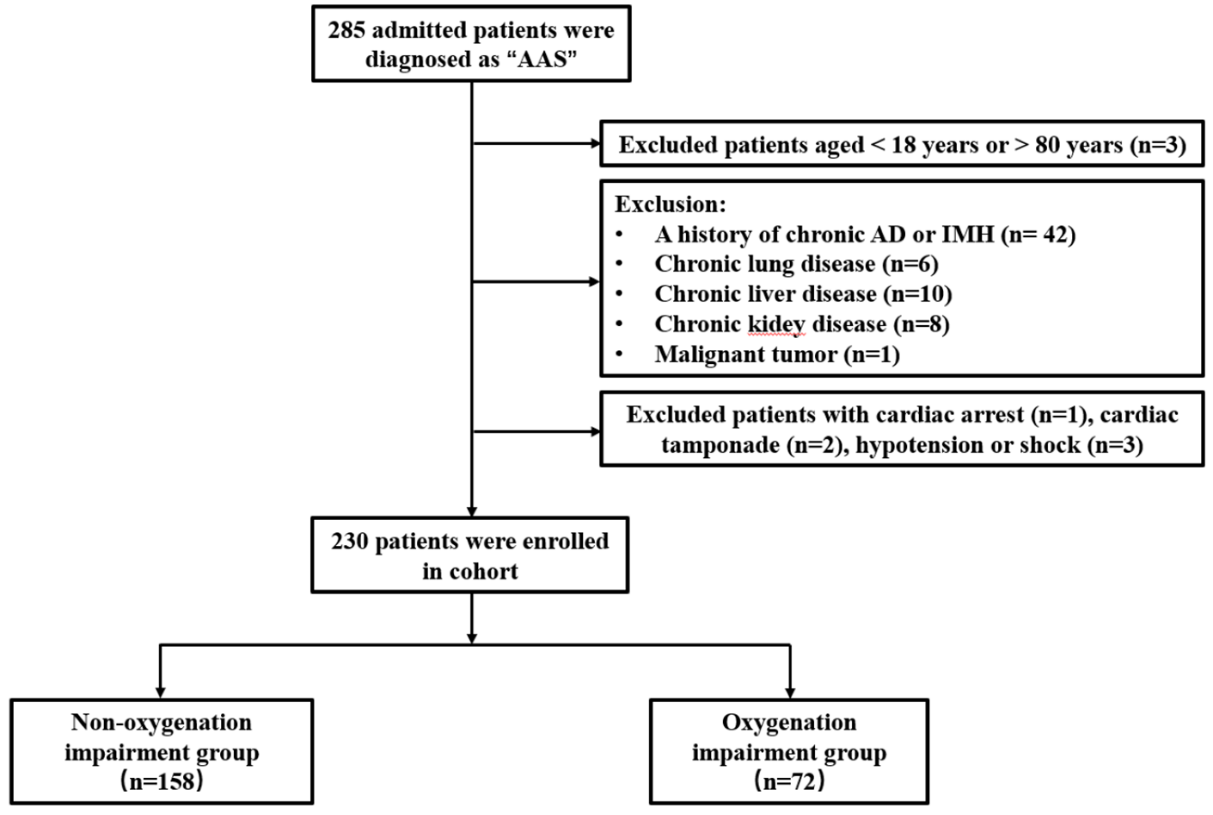


Supplementary Figure 5. Flow diagram of AAS patients in the retrospective study. AAS: acute aortic syndrome.
