# Supplementary material for: The association between body mass index and risk of preoperative oxygenation impairment in patients with the acute aortic syndrome
Source: Front Endocrinol (Lausanne). 2022 Nov 17;13:1018369. doi: 10.3389/fendo.2022.1018369 (PMC9712723; doi:10.3389/fendo.2022.1018369)
Supplement: Supplementary file 2 [file Table_1.docx]

**Supplementary Table 1.** Characteristics of included studies on BMI and the risk of AAS with preoperative oxygenation impairment.

| First author | Year | Country | Ethnicity | Study size, sex (M/F), number of cases | Diagnostic criteria of preoperative oxygenation impairment | BMI | OR  (95% CI) | Adjustment factors | NOS score |
| --- | --- | --- | --- | --- | --- | --- | --- | --- | --- |
| Xuzhou Duan | 2016 | China | Asian | 70, (43/27), 21 | P_aO2_ / F_iO2_ ≤ 200 | Continuous | 1.15 (0.74-1.79) | Age, gender, CRP, IL-6, D-dimer | 7 |
| Xudong Pan | 2018 | China | Asian | 130, (98/32), 70 | P_aO2_ / F_iO2_ ≤ 300 | Continuous | 1.31 (1.09-1.56) | Gender, history of smoking, aortic regurgitation, TNF-α, TFPI, IL-10, TSOD, PLC | 7 |
| Zhiyun Xu | 2018 | China | Asian | 172, (135/37), 77 | P_aO2_ / F_iO2_ ≤ 200 | Continuous | 1.18 (0.94-1.48) | WBC, neutrophils, D-dimer, CRP, IL-6, involve celiac trunk, involve mesenteric artery, involve renal artery | 8 |
| Dilixiati Siti | 2018 | China | Asian | 143, (unknown), unknown | acute lung injury | < 25.65  ≥ 25.65 | 1.00  5.89 (2.12-16.40) | Unknown | 6 |
| Zijian Guo | 2019 | China | Asian | 505,(395/110, 235 | P_aO2_ / F_iO2_ ≤ 300 | Continuous | 1.06 (1.01-1.11) | Unknown | 7 |
| Yudai Tamura | 2021 | Japan | Asian | 224, (140/84), 53 | P_aO2_ / F_iO2_ ≤ 200 | < 25  ≥ 25 | 1.00  2.36 (1.13-4.97) | Male, current smoker, WBC > 15,000/μL, patent false lumen, Peak CRP, D-dimer | 8 |

M/F: male/female; BMI: body mass index; OR: odds ratio; NOS: Newcastle-Ottawa Scale; P_aO2_ / F_iO2_: arterial oxygen tension / inspiratory oxygen fraction; CRP: C reactive protein; IL-6: interleukin-6; TNF-α: tumor necrosis factor α; TFPI: tissue factor pathway inhibitor; IL-10: interleukin-10; TSOD: total superoxide dismutase; PLC: platelet count; WBC: white blood cell.

**Supplementary Table 2.** Subgroup analysis of BMI and the risk of AAS with preoperative oxygenation impairment in the meta-analysis.

|  |  | Fixed-effect model | |  | Random-effect model | |  | Heterogeneity | |
| --- | --- | --- | --- | --- | --- | --- | --- | --- | --- |
|  | N | OR (95%Cl) | *p* |  | OR (95%Cl) | *p* |  | *I*^2^% | *Q* |
| All study | 6 | 1.09(1.04-1.14) | <0.001 |  | 1.30(1.05-1.60) | 0.015 |  | 75.6 | 0.001 |
| Stanford classification |  |  |  |  |  |  |  |  |  |
| Type A | 4 | 1.08(1.03-1.13) | 0.001 |  | 1.30(1.05-1.60) | 0.027 |  | 47.6 | 0.126 |
| Type B | 2 | 3.23(1.77-5.89) | <0.001 |  | 3.47(1.43-8.42) | 0.006 |  | 50.4 | 0.156 |
| Different data forms for BMI |  |  |  |  |  |  |  |  |  |
| categorical | 2 | 3.23(1.77-5.89) | <0.001 |  | 3.47(1.43-8.42) | 0.006 |  | 50.4 | 0.156 |
| continuous | 4 | 1.08(1.03-1.13) | 0.001 |  | 1.15(1.02-1.29) | 0.027 |  | 47.6 | 0.126 |
| Sample size |  |  |  |  |  |  |  |  |  |
| < 150 | 3 | 1.34(1.14-1.58) | 0.001 |  | 1.64(1.05-1.60) | 0.079 |  | 76.7 | 0.014 |
| ≥ 150 | 3 | 1.07(1.02-1.12) | 0.005 |  | 1.17(0.94-1.45) | 0.157 |  | 61.8 | 0.073 |

BMI: body mass index; AAS: acute aortic syndrome; OR: odds ratio; CI: confidence interval.
